# Impact Ionization Induced by Terahertz Radiation in HgTe Quantum Wells of Critical Thickness

**DOI:** 10.1007/s10762-020-00690-6

**Published:** 2020-04-06

**Authors:** S. Hubmann, G.V. Budkin, M. Urban, V.V. Bel’kov, A.P. Dmitriev, J. Ziegler, D.A. Kozlov, N.N. Mikhailov, S.A. Dvoretsky, Z.D. Kvon, D. Weiss, S.D. Ganichev

**Affiliations:** 1 Terahertz Center, University of Regensburg, 93040 Regensburg, Germany; 2 Ioffe Institute, 194021 St. Petersburg, Russia; 3 Rzhanov Institute of Semiconductor Physics, 630090 Novosibirsk, Russia

**Keywords:** Terahertz, Nonlinearities, Impact ionization, HgTe quantum wells

## Abstract

We report on the observation of terahertz (THz) radiation induced band-to-band impact ionization in HgTe quantum well (QW) structures of critical thickness, which are characterized by a nearly linear energy dispersion. The THz electric field drives the carriers initializing electron-hole pair generation. The carrier multiplication is observed for photon energies less than the energy gap under the condition that the product of the radiation angular frequency *ω* and momentum relaxation time *τ*_l_ larger than unity. In this case, the charge carriers acquire high energies solely because of collisions in the presence of a high-frequency electric field. The developed microscopic theory shows that the probability of the light-induced impact ionization is proportional to $\exp (-{E_{0}^{2}}/E^{2})$, with the radiation electric field amplitude *E* and the characteristic field parameter *E*_0_. As observed in experiment, it exhibits a strong frequency dependence for *ωτ* ≫ 1 characterized by the characteristic field *E*_0_ linearly increasing with the radiation frequency *ω*.

## Introduction

Impact ionization across the band edges and its inverted process - Auger recombination - and impact ionization of impurities are the most important autocatalytic processes in semiconductors. They have been studied extensively not only because of fundamental interest in these nonlinear phenomena but also due to their great practical importance for IMPATT diodes (impact ionization avalanche transit time) [1], high-efficiency solar cells [2], and photodetectors with internal amplification like avalanche photodiodes, particularly useful in the case of fiber-optic communication systems [3]. Aside from being excited by a *dc* electric field like the aforementioned processes, impact ionization can also be excited by the *ac* electric field of THz radiation. Such a process has been observed first in bulk InSb crystals and was called light impact ionization [4, 5]. With the development of high-power THz laser systems like molecular lasers, free-electron lasers, and Ti:Sapphire-based THz systems, there has been a steady increase on experimental and theoretical research interest in the field of THz radiation-induced impact ionization, carrier multiplication, and nonperturbative nonlinearities in three- and two-dimensional semiconductor systems [6–24], for reviews see [25, 26]. Recently it has been shown that impact ionization and Auger recombination processes can also be efficiently excited and probed by THz radiation in graphene [27–31]. Interband carrier-carrier scattering such as impact ionization- and/or Auger-type processes in graphene is of particular importance. Because of the peculiar linear dispersion in Dirac materials and the conservation laws, they are allowed only when the momenta of all the particles involved in the ionization/recombination are co-linear. However, it has been shown that these processes becomes non-vanishing either due to the extent of a small difference between the carrier dispersion from the linear one (e.g., trigonal warping) or due to many body effects such as plasmon-assisted processes or additional scattering by an impurity or phonon [32–40]. A suppression of the Auger recombination has also been addressed for HgTe-based QW structures with symmetric dispersion laws in conduction and valence bands [41] and for massless Kane fermions in HgCdTe-based materials [42]. This suppression has been used to obtain band-band population inversion and stimulated THz emission, which, because of the efficient nonradiative Auger recombination, can not be achieved in conventional narrow band semiconductors. When approaching the critical thickness in HgTe/HgCdTe QWs, the band structure gets almost linear [43], i.e., similar to that of graphene, but characterized by electron spin instead of pseudo-spin. The linear dispersion in HgTe/HgCdTe QWs with critical thickness has been demonstrated in transport [44] and THz experiments [45–47].

Here, we show that excitation of such QWs by intense THz radiation results in an efficient impact ionization process. Applying monochromatic radiation with frequencies from 0.6 to 2 THz, we observed a photoconductivity signal rising superlinearly with the radiation intensity *I*. The photoconductivity is caused by the generation of electron-hole pairs and, in a large range of radiation intensities, varies as $\exp (-{E_{0}^{2}}/E^{2})$, where *E* is the radiation electric field amplitude $E\propto \sqrt I$ and *E*_0_ is the characteristic field parameter. Furthermore, it shows a strong frequency dependence decreasing with the frequency increase. The observed field and frequency dependencies indicate that the generation of electron-hole pairs is caused by the light-induced impact ionization in high-frequency electric fields. As shown in ref. [48], light-induced impact ionization is divided into two regimes: (i) the quasi-static, in which the angular radiation frequency *ω* = 2*πf* is much lower than the reciprocal momentum relaxation time *τ*^− 1^ and the ionization takes place within a half period of the field, and (ii) the high-frequency regime with *ω* ≫ *τ*^− 1^, in which carriers acquire the ionization energy due to collisions. In our experiments, $\omega \gtrsim \tau ^{-1}$ which corresponds to the latter regime characterized by strong frequency dependence. We developed a theory considering impact ionization for the real band structure of HgTe QWs with thickness close to the critical one. The theory describes both the quasi-static and high-frequency regimes. It describes all experimental findings well and shows that the observed nonlinear photoconductivity is caused by the latter regime also known as light impact ionization.

## Samples and Methods

**Fig. 1:**
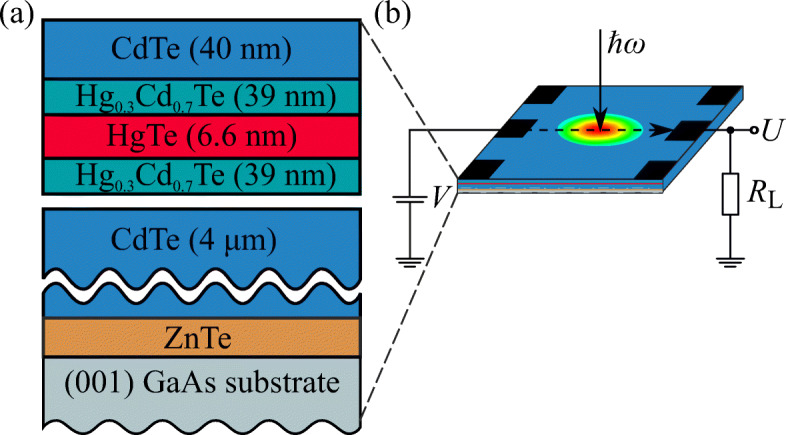
**a** Structure composition. **b** Sketch of the setup used for the photoconductivity measurements

**Fig. 2:**
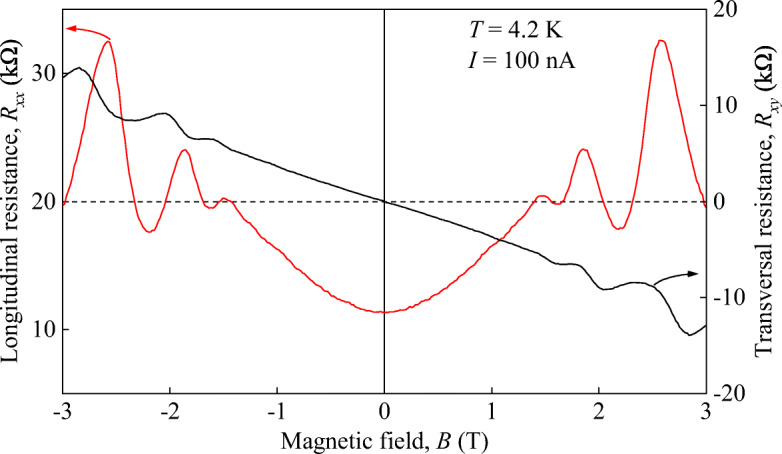
Magnetotransport data obtained at liquid helium temperature with a current of 100 nA. The red line shows the longitudinal resistance *R*_*xx*_ while the black line shows the transversal resistance *R*_*xy*_

The samples studied in this work are HgTe/HgCdTe QWs grown by molecular beam epitaxy on (001)-oriented GaAs substrates by an analogous procedure as described in Ref. [49]. The 6.6-nm-wide QWs were surrounded by two 39-nm-thick Hg_0.3_Cd_0.7_Te barriers, see Fig. 1a. In order to relax strain stemming from the lattice mismatch between the GaAs substrate and HgCdTe, a 30-nm-thick ZnTe layer and a 4-μm-thick CdTe buffer layer were grown in between. This structure composition leads to a almost linear energy spectrum [43–46]. Our *k* ⋅ *p* calculations presented below show that in the structure, a small band gap of about *ε*_g_ = 4.5 meV should be present. The sample size was 5 × 5 mm^2^ in a van-der-Pauw sample geometry, see Fig. 1b. Ohmic contacts were fabricated by indium soldering to make photoconductivity and magnetotransport measurements possible. Applying magnetotransport measurement, we obtained a carrier density of 1.7 × 10^11^ cm^− 2^ and a mobility of 5700 cm^2^/Vs, see Fig. 2.

To apply a high-frequency electric field, we used the THz radiation from an optically pumped high-power pulsed molecular gas THz laser [50–52]. This laser system features several frequency lines between 0.6 and $3.3~\text {THz} \left (2.5 \text { to } 14~\text {meV}\right )$ with a pulse duration of about 100 ns, a repetition rate of 1 Hz, and a gaussian beam shape. The latter has been measured by a pyroelectric camera [53]. Using a parabolic mirror, the beam was focused onto the sample with a spot diameter of about 2.5 mm. The time structure of THz pulses was controlled by a fast room temperature photon-drag detector [54]. The samples were placed in an optical temperature-regulated continuous flow cryostat with *z*-cut crystal quartz windows. The measurements have been carried out in a temperature range from 4.2 to 90 K. All experiments were performed illuminating the sample with the THz laser radiation under normal incidence. In order to vary the laser radiation intensity, a crossed polarizer setup was used: First, the linearly polarized radiation passed a wire grating polarizer, which was rotated to modify the radiation intensity. Then a second polarizer at a fixed position ensured a fixed output polarization.

The *dc* photoconductivity was measured using the setup shown in Fig. 1b. A *dc* bias voltage *V* = 0.3 V was applied and the voltage drop *U* in response to the laser pulse was measured across a load resistor *R*_L_. The photoconductivity signal can be separated from possible photocurrent contributions by subtracting signals detected for negative and positive bias voltages and dividing by 2, since the photocurrent contributions are not sensitive to the bias voltage in contrast to the photoconductivity signal.

## Results

First, we discuss the data for radiation with photon energies $\hbar \omega $ smaller than the band gap *ε*_g_ ≈ 4.5 meV.

**Fig. 3:**
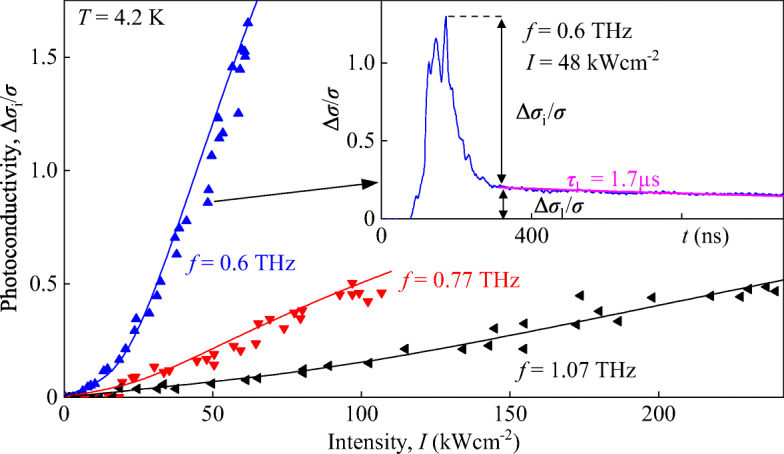
Intensity dependence of the photoconductivity signal Δ*σ*_i_/*σ* for frequencies of 0.6 (blue triangles), 0.77 (red triangles), and 1.07 THz (black triangles). Solid lines present the fit according to Eq. 1 with the fitting parameters *A*, *B*, and *I*_0_. Note that for all frequencies $\hbar \omega <\varepsilon _{\text {g}}$ is valid. Inset shows the typical kinetics of the photoconductivity pulse for a frequency of 0.6 THz and an intensity of 48 kW cm^− 2^. The magenta line shows a fit of the exponential decay according to ${\Delta }\sigma _{\text l}/\sigma \propto \exp \left (-t/\tau _{\text l} \right )$

The inset in Fig. 3 shows the photoconductive response observed applying radiation with a frequency of *f* = 0.6 THz to the sample cooled down to liquid helium temperature. The detected signal temporal shape, being characteristic for all frequencies and all studied temperatures, consists of two parts characterized by different response times and relative amplitudes. The first part (Δ*σ*_i_/*σ*) has a response time in the range of several tens of nanoseconds. The second part (Δ*σ*_l_/*σ*) has a substantially longer response time, being in the microsecond range. While at high intensities, the contribution Δ*σ*_i_/*σ* yields the highest signal at low intensities the situation changes and Δ*σ*_l_/*σ* dominates. In the following, we will first focus on Δ*σ*_i_/*σ*, which, as we show below, is caused by light-induced impact ionization. Due to substantial difference in the signal kinetic, this contribution can be easily extracted from the total signal.

The intensity dependencies of Δ*σ*_i_/*σ* for three frequencies and *T* = 4.2 K are shown in Fig. 3. The data demonstrate a superlinear dependence of the signal on the radiation intensity. The data are fitted well by

1 $$ \frac{\Delta\sigma_{\text i}}{\sigma}=A\cdot I+B\cdot\exp\left( -\frac{I_{0}}{I}\right)=A_{\text E}\cdot E^{2}+B\cdot\exp\left( -\frac{{E_{0}^{2}}}{E^{2}}\right) , $$

**Fig. 4:**
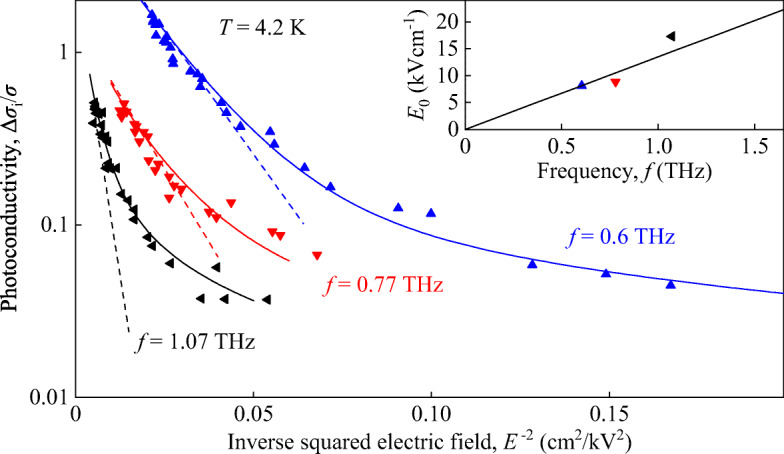
Dependency of Δ*σ*_i_/*σ* on the inverse squared electric field obtained for frequencies of 0.6 (blue triangles), 0.77 (red triangles), and 1.07 THz (black triangles). The data are presented in a half-logarithmic plot. Solid lines show the fits according to Eq. 1. Dashed lines show fits after exponential term in right hand side of Eq. 1 which gives the same field dependence as the theoretical Eq. 16. Inset shows the dependence of the fit parameter *E*_0_ on frequency. Solid line is a linear fit after Eq. 16.

Here, *I* = (*E*^2^ ⋅ *n*_*ω*_/(2*Z*_0_) is the radiation intensity, *E* is the radiation electric field, *n*_*ω*_ is the refractive index, *Z*_0_ is the vacuum impedance, $I_{0}=({E^{2}_{0}}\cdot n_{\omega }/(2Z_{0})$ is the characteristic intensity, *E*_0_ is the characteristic electric field, and *A*_E_ = *A* ⋅ *n*_*ω*_/(2*Z*_0_). The fits shown in Fig. 3 are obtained using fitting parameters *I*_0_, *A*, and *B*. An important observation is that the nonlinearity is defined by the characteristic intensity $I_{0}\propto {E_{0}^{2}}$ and that lowering the radiation frequency results in a substantial increase of the signal amplitude. As we show below, the exponential part of the right-hand side in Eq. 1 with *E*_0_ ∝ *ω* coincides with that obtained from the theoretical examination of light-impact ionization, see Eq. 16. Re-plotting the data in a half-logarithmic plot as a function of the inverse squared electric field *E*^− 2^, Fig. 4, we obtain that at high radiation electric fields, the exponential term describes well our results. As it is shown in the inset in Fig. 4, scaling of *E*_0_ with *ω*, being characteristic for the light-induced impact ionization [4, 24], is also detected. At low intensities, however, we find a deviation from this behavior and the signal is determined by the first term in the left-hand side of Eq. 3.

**Fig. 5:**
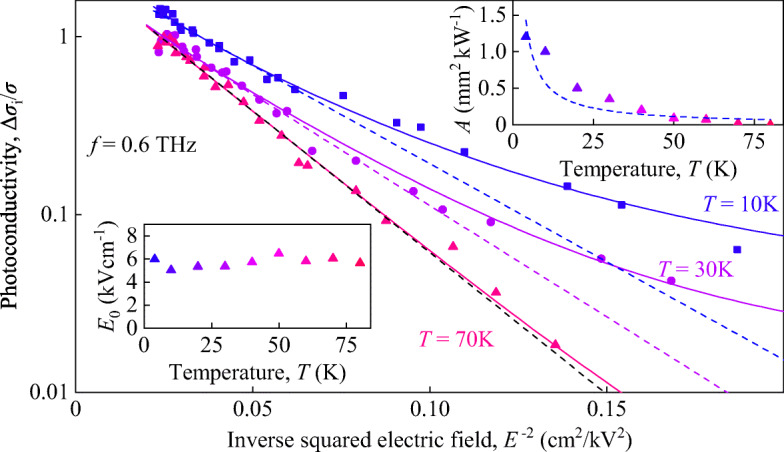
Photoconductivity Δ*σ*_i_/*σ* as a function of the inverse squared electric field *E*^− 2^ measured for three temperatures: 10 K (blue squares), 30 K (purple circles) and 70 K (pink triangles). The data are obtained at 0.6 THz and are presented in a half-logarithmic plot. Solid lines show fits according to Eq. 1. Dashed lines show fits according to the exponential term in right hand side of Eq. 1 which gives the same field dependence as the theoretical Eq. 16. Insets show temperature dependencies of ${E_{0}^{2}}$ and parameter *A*

Rising temperature results in a slight increase of the characteristic electric field *E*_0_ and a substantial decrease of the fitting parameter *A* defining the first term in Eq. 1. Figure 5 shows the data obtained for three different temperatures at radiation frequency *f* = 0.6 THz indicating that already at *T* = 70 K, the exponential term in Eq. 1 dominates the photoconductivity in the whole range of the radiation intensity. Temperature evolution of ${E_{0}^{2}}$ and parameter *A* are shown in the insets in Fig. 5.

**Fig. 6:**
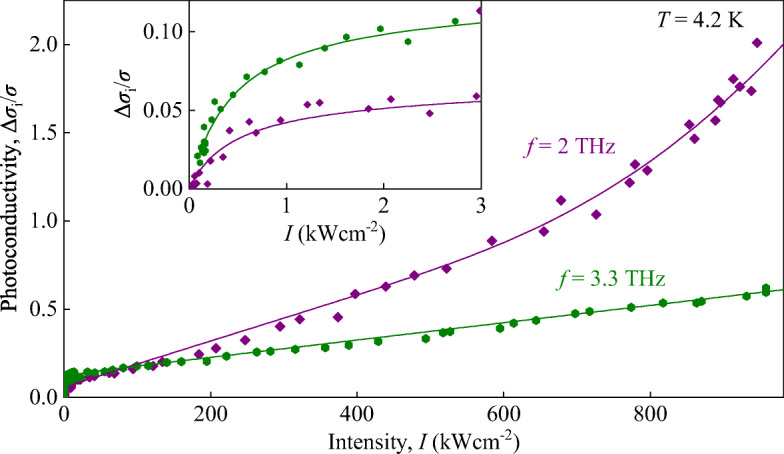
Intensity dependences of Δ*σ*_i_/*σ* at 2 (violet diamonds) and 3.3 THz (green hexagons). Solid lines present fits according to Eq. 2. Note that for both frequencies $\hbar \omega >\varepsilon _{\text g}$ is valid. The inset shows a zoom-in for low intensities

Now, we discuss the photoconductivity obtained for photon energies higher than the energy gap, $\hbar \omega >\varepsilon _{\text g}$. An increase of the photon energy qualitatively changes the intensity dependence of Δ*σ*_i_/*σ*: At highest frequency used (*f* = 3.3 THz), we observed instead of superlinear behavior that the signal saturates with rising intensity, see green triangles and line in Fig. 6. Saturation with increasing radiation intensity is also clearly seen for *f* = 2 THz; however, for these frequencies, a superlinear behavior shows up and becomes dominant at high intensities, see violet diamonds and line Fig. 6. Our experiments show that for $\hbar \omega >\varepsilon _{\text g}$, an additional term describing the saturation of the photoconductivity should be added to Eq. 1 and the overall intensity dependence is given by

2 $$ \frac{\Delta\sigma_{\text i}}{\sigma}=C\frac{I}{1+I/I_{S}}+A\cdot I+B\cdot\exp\left( -\frac{I_{0}}{I}\right) $$

with the prefactor *C* and the saturation intensity *I*_*S*_ used to describe the signal saturation.

**Fig. 7:**
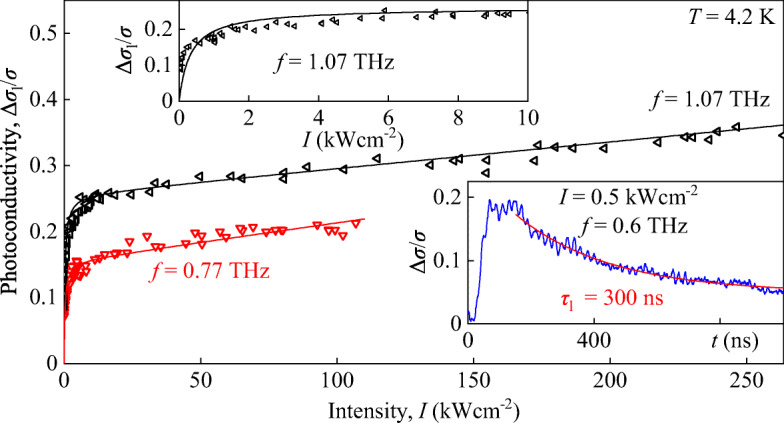
Intensity dependence of the slow component of the photoconductivity signal Δ*σ*_l_/*σ*. The data are shown for frequencies of 0.77 (red triangles) and 1.07 THz (black triangles). Solid lines present fits according to Eq. 3. The left inset shows a zoom-in for low intensities. The right inset shows the typical kinetics of the photoconductivity pulses obtained for *f* = 0.6 THz and *I* = 0.5 kWcm^− 2^. The red line shows an exponential decay fit according to ${\Delta }\sigma /\sigma \propto \exp \left (-t/\tau _{\text l} \right )$

Finally, we describe the slow photoconductive signal component Δ*σ*_l_/*σ*. For the temperature of 4.2 K and at low intensities, the slow response dominates the signal and is characterized by a time constant of 0.3 μs, see inset in Fig. 7. Figure 7 shows the corresponding intensity dependence for frequencies 0.77 and 1.07 THz. The data reveal that the slow component of the signal at low intensity increases linearly with *I* and saturates at high intensities. The data are fitted according to

3 $$  \frac{\Delta\sigma_{\text l}}{\sigma}=C_{l}\frac{I}{1+I/I_{l,S}}+A_{l}\cdot I $$

with the fitting parameters *A*_*l*_, *I*_*l*,*S*_, and *C*_*l*_. Because of the saturation, at high intensity, the time dynamics of the signal gets substantially slower reaching time constants of about 2 μs, see inset in Fig. 3. Rising the temperature, Δ*σ*_l_/*σ* vanishes for *T* > 20 K (not shown). This fact together with the slow kinetic of the photoconductive signal indicates that it is caused by ionization of impurities in the HgTe QW. Indeed, at high temperatures, impurities get thermally ionized and consequently, the photosignal vanishes. Furthermore, at high radiation intensities, impurities become completely ionized resulting in the signal saturation as detected in our experiment. Extrinsic photoconductivity and its saturation are well-known processes and, therefore, their consideration is out of scope of our paper.

## Discussion and Theory

Our measurements show that THz excitation of HgTe QWs with almost linear energy dispersion leads to a photoconductivity showing a strongly nonlinear dependence on the radiation intensity. Under all conditions, we observed positive photoconductivity corresponding to a decrease of the sample resistance due to illumination. While the fast photoconductive signal rises linearly with the radiation intensity at low intensities and small photon energies, it shows a superlinear behavior at high intensities, see Fig. 3. The former signal is attributed to the bolometric effect caused by Drude-like absorption resulting in electron gas heating and consequently, in the change of carrier mobility. The mechanisms of this effect are well known [25] and are out of scope of the present paper. At high intensity, the signal kinetics correspond to the recombination time of photogenerated electron-hole pairs [55]. The exponential growth of the signal ${\Delta }\sigma _{\text i}/\sigma \propto \exp (-{E_{0}^{2}}/E^{2})$, detected at high intensities, together with its frequency dependence given by ${E_{0}^{2}}\propto \omega ^{2}$, see Figs. 4 and 5, provide an indication that it is caused by band-to-band light-induced impact ionization [4–7, 24]. These results are in focus of our work. Below, we present the theory of the impact ionization caused by the electric field of THz radiation and show that it describes well our findings.

For the theory of light-induced impact ionization, the knowledge of the band structure is crucially needed. The electron spectrum of 6.6 nm (001)-oriented HgTe QWs is calculated within the eight-band *k* ⋅ *p*-model. The effective Hamiltonian takes into account conduction, valence, and spin-orbit split-off bands and is taken from Ref. [56].

**Fig. 8:**
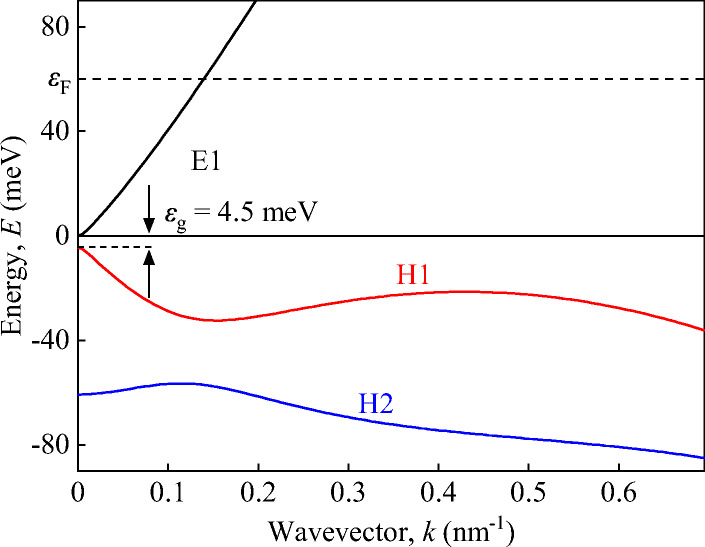
Calculated energy dispersion of the structures used in this study. The energy dispersion is almost linear around *k* = 0 with a band gap of *ε*_g_ = 4.5 meV. The Fermi energy is *ε*_F_ ≈ 60 meV

**Fig. 9:**
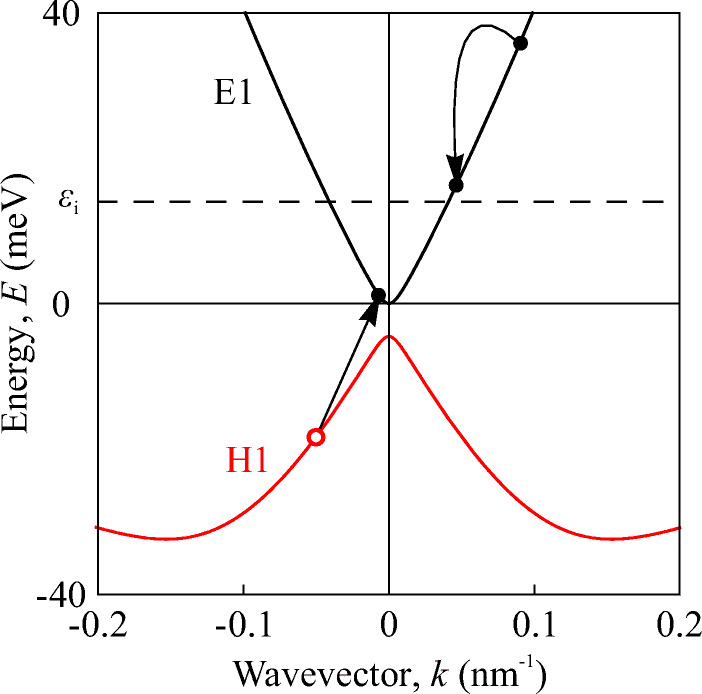
Sketch of ionization process. Arrows show the transitions of charge carries: An electron with high kinetic energy in the conduction band knocks the electron in the valence band by transferring part of its kinetic energy. As a result of such a process an electron-hole pair is created

Figure 8 shows the electronic band dispersion calculated for the structure investigated in this work. The calculations show that the thickness of the QW is close to the critical width and that the band gap *ε*_g_ = 4.5 meV is small. The figure reveals that the energy spectrum of electrons is close to linear, while the spectrum of holes at large wavevectors *k* significantly differs and has a complex dependence on the wavevector. The minimum kinetic energy *ε*_*i*_ required for a conducting electron to lift a valence band electron into the conduction band, creating electron-hole pair is found from energy and momentum conservation for the dispersion shown in Fig. 8. The illustration of the act of ionization is shown in Fig. 9. Our numerical calculation shows that the threshold energy of impact ionization *ε*_*i*_ for this band structure is approximately equal to 14 meV, which, under our experimental conditions, is more than four times smaller than the Fermi energy *ε*_*F*_ ≈ 60 meV. Impact ionization for *ε*_g_ < *ε*_*F*_ was considered in [24] showing that under these conditions, the electron-hole pair generation is limited by the small number of free low-energy states in the conduction band. Therefore, in the ionization process heating of the electron gas is required in order to deplete occupied states in the low-energy region rather than increase the number of “hot” electrons.

In the following, we assume that the main mechanism of electron momentum relaxation is scattering by impurities, while the energy relaxation of electrons heated by radiation is due to the interaction with optical phonons. We also suppose that the electron heating on the one hand is strong enough, so that the initially step-like Fermi distribution function is smoothed and changes slowly on the energy scale of the optical phonon. On the other hand, we consider that heating is not too strong for the average electron energy to become much higher than the initial Fermi level. These assumptions allow us to use the results of [24].

For electron energies *ε* lower than the energy of optical phonons *ε*_0_, the electron distribution function is almost independent of energy and is equal to its value at *ε* = *ε*_0_. In the region of higher energies, the distribution function is given by [24]

4 $$  f_{0}(\varepsilon)=\frac{1}{1+\exp\left[- L(\varepsilon)\right]} , \quad L(\varepsilon)=\int\limits_{\varepsilon} ^{\varepsilon_{E}}\frac{\varepsilon_{0}}{D(\varepsilon^{\prime})\tau_{\text{ph}}(\varepsilon^{\prime})} d\varepsilon^{\prime} , $$

where $\tau _{\text {ph}}^{-1}(\varepsilon )$ is the rate of electron scattering by optical phonons, *D*(*ε*) is the diffusion coefficient of electrons in energy space caused by their diffusive motion in real space in the field of an electromagnetic wave and *ε*_*E*_ is determined by the normalization by the density

5 $$  n=\int\limits_{0}^{\infty}f_{0}(\varepsilon) g(\varepsilon) d\varepsilon. $$

The diffusion coefficient *D*(*ε*) has the form

6 $$  D(\varepsilon)=\frac{e^{2} E^{2} v^{2}(\varepsilon)}{4 \omega^{2} \tau_{i}(\varepsilon)} , $$

and the rate $\tau _{\text {ph}}^{-1}(\varepsilon )$ for the Fröhlich mechanism of electron-phonon interaction (see Ref. [57]) in quantum wells is given by

7 $$  \frac{1}{\tau_{\text{ph}}(\varepsilon)}= \frac{4 \pi^{2} \varepsilon_{0} e^{2} g(\varepsilon)}{\overline{\epsilon}p(\varepsilon)} , $$

where $1/\overline {\epsilon }=1/{\epsilon _{\infty }}-1/\epsilon _{0}$, $\epsilon _{\infty }$ and *𝜖*_0_ are high- and low- frequency dielectric permittivities, *g*(*ε*) is the density of states, *p*(*ε*) is the electron momentum, *v*(*ε*) is electron velocity, and *τ*_*i*_(*ε*) is the momentum relaxation time due to scattering by impurities. We note that the model assumes ${\varepsilon _{i}\lesssim \varepsilon _{0},\varepsilon }$.

For the studied samples, the spectrum of the conduction band electrons is close to linear, *ε* = *v*_*F*_*p*, where *v*_*F*_ is the Fermi velocity. Thus, one obtains $g(\varepsilon )=\varepsilon /\pi \hbar ^{2} {v_{F}^{2}}$ and *p*(*ε*) = *ε*/*v*_*F*_. Hence, the rate of electron scattering by phonons is given by

8 $$ \frac{1}{\tau_{\text{ph}}(\varepsilon)}=\frac{4 \pi \varepsilon_{0} e^{2}}{v_{F}\overline{\epsilon}\hbar^{2}} . $$

As addressed above, under experimental conditions, initially neutral donors are ionized by the light wave, so that electrons are scattered by charged impurities. In the case of a linear spectrum, the momentum relaxation time is given by $\tau _{i}^{-1}(\varepsilon )=\tau _{iF}^{-1}(\varepsilon _{F}/\varepsilon )$, which for *ωτ*_*i*_(*ε*) ≫ 1 leads to

9 $$  D(\varepsilon)=\frac{e^{2} E^{2} {v_{F}^{2}} \varepsilon_{F}}{4 \omega^{2} \tau_{iF} \varepsilon} $$

Using Eqs. 6 and 9 in Eq. 4, we obtain

10 $$  L(\varepsilon)=\frac{{\varepsilon_{E}^{2}}-\varepsilon^{2}}{\tilde{\varepsilon}^{2}} , $$

here, we introduce the notation

11 $$  \tilde{\varepsilon}^{2}= \frac{\overline{\epsilon} {v_{F}^{3}} \hbar^{2} \varepsilon_{F} E^{2}}{8 \pi \varepsilon_{0} \tau_{iF} \omega^{2}} . $$

The distribution function Eq. 4 can be written as

12 $$  f_{0}(\varepsilon)= \frac{1}{1+{\Lambda}\exp(\varepsilon^{2}/\tilde{\varepsilon}^{2})} , $$

where ${\Lambda }=\exp (-{\varepsilon _{E}^{2}}/\tilde {\varepsilon }^{2})$. Taking into account both the normalization by density, Eq. 5, and distribution function, Eq. 12, we obtain

13 $$ {\Lambda}=\frac{1}{\exp(2\pi n\hbar^{2} {v_{F}^{2}}/\tilde{\varepsilon}^{2})-1} . $$

As previously mentioned, the rate of electron-hole pair generation is proportional to the number of unoccupied states in the low-energy region *ε* ≪ *ε*_*E*_ of the conduction band, i.e., defined by the function *ρ*(*ε*) = 1 − *f*_0_(*ε*), which, according to Eq. 12, is given by the expression ${\rho (\varepsilon )\approx {\Lambda } \exp (\varepsilon ^{2}/\tilde {\varepsilon }^{2})}$. The condition *ρ*(*ε*) ≪ 1 at low energy region implies that Λ ≪ 1, and thus

14 $$  \rho(\varepsilon)\approx \exp[-(2\pi n \hbar^{2} {v_{F}^{2}}-\varepsilon^{2})/\tilde{\varepsilon}^{2}] . $$

The total number of excited electron-hole pairs in the sample at a given radiation intensity depends on the number of unoccupied states *ρ*(*ε*) as well as on the probability of impact ionization and on the recombination rate of electron-hole pairs. The two latter quantities are unknown and as a result the exact region of energies that makes the dominant contribution to rate of generation *W* cannot be determined. However, the knowledge of this range is in fact not so important for the calculation of the functional dependence of *W* on the radiation electric field and frequency. This is because the value of *W* is proportional to the square of the exponential term *ρ*(*ε*) (the power two arises because two electrons should be able to occupy the unoccupied states in the low-energy region). Denoting the characteristic energy corresponding to the low-energy region from Eq. 14 by *ε*_*c*_, we obtain

15 $$  W \propto \rho^{2}(\varepsilon_{c})=\exp[-2(2\pi\hbar^{2} {v_{F}^{2}}-{\varepsilon_{c}^{2}})/\tilde{\varepsilon}^{2}]. $$

Taking into account Eq. 10 for $\tilde {\varepsilon }$ allows us to get the field and frequency dependencies of the number of excited electrons, which is given by

16 $$  W\propto \exp(-{E_{0}^{2}}/E^{2}) , \quad {E_{0}^{2}}\propto \omega^{2} . $$

The frequency dependence in Eq. 16 is obtained for the relevant experimental condition *ωτ*_*i*_(*ε*) ≫ 1. Generally, the dependence of the distribution function on frequency and, as a consequence, the dependence of the pair generation rate Eq. 16 on *ω* are solely determined by the frequency variation of *D*(*ε*) defined by Eq. 9. For an arbitrary value of *ωτ*_*i*_(*ε*), according to Eq. 6, the diffusion coefficient of electrons in energy space is given by

17 $$ D(\varepsilon)=\frac{e^{2} E^{2} \tau_{iF} \varepsilon/\varepsilon_{F}}{4(1+\omega^{2} \tau_{iF}^{2} \varepsilon^{2}/{\varepsilon_{F}^{2}})}. $$

This equation shows that for the condition *ωτ*_*i*_(*ε*) ≪ 1, the dependence of *D*(*ε*) vanishes, and thus the distribution function is also independent of frequency. Carrying out the same calculation as above, we again obtain an expression for the pair generation rate Eq. 16; however, in this case, *E*_0_ does not depend on the frequency *ω*. Finally, we note that for the case of weak heating, when the above assumption of a slowly changing distribution function on the optical phonon energy scale is not fulfilled, the calculation can not be solved analytically. It can only be stated that, similarly to the case of a static electric field [48, 58], the number of excited pairs is determined by the exponent $\exp (-E_{1}/E)$, where *E*_1_ is independent of the frequency for *ωτ*_*i*_(*ε*) ≪ 1.

Comparing the theoretical Eq. 16 with experiment, we see that it describes the observed superlinear intensity dependence of the generation rate of electron-hole pairs at high intensities well. Indeed, experiments show that ${\Delta }\sigma _{\text i}/\sigma \propto \exp (-{E_{0}^{2}}/E^{2})$ and $ {E_{0}^{2}}\propto \omega ^{2}$ agrees with Eq. 16, see Figs. 4 and 5. The dependence of the photoconductive response on radiation intensity is similar to the one obtained under the same conditions for HgTe QWs with *L*_*w*_ = 5.7 nm, ref. [24]. Despite that the bandgaps of the structures differ by a factor of 4, we attribute this similar behavior to the fact that the Fermi energy in both structures is much higher than the band gap and, as a result, quantum transitions, leading to impact ionization, mainly involve electrons located in the linear region of the energy spectrum.

The observed increase of ${E_{0}^{2}}$ with increasing temperature indicates the reduction of the impact ionization rate, see left inset in Fig. 5 and is also in line with the above theory. Indeed, rising the temperature results in an increase of energy losses due to emission of phonons and, subsequently, in the reduction of radiation-induced electron gas heating. Note that the same temperature behavior was previously reported for light-induced impact ionization of HgTe QWs with *L*_*w*_ = 5.7 nm [24].

While our paper is aimed to the light-induced impact ionization, we briefly discuss the saturation of the photoconductivity signal observed for the fast photoconductivity response excited by $\hbar \omega >\varepsilon _{g}$, see Fig. 6. In this case, radiation absorption due to inter-band optical transitions is also possible, in addition to Drude-like transition. At low intensities, the inter-band transitions play almost no role because the final states of these transitions are lying below the Fermi energy and thus, these states are occupied. At high intensities, however, electron gas heating, discussed above, depletes occupied states in the low-energy region of the conduction band and direct band-to-band transitions contribute to the photoconductivity signal. The interplay of the signal components caused by Drude absorption, direct band-to-band transitions, and impact ionization causes a complex intensity dependence of the total signal, see Eq. 2. We attribute the two first terms on the right hand side of this equation to direct optical transitions and Drude absorption. The saturation of the fundamental absorption at high intensities is a well-known process. Recently, it has been demonstrated that Drude-like transitions under strong electron gas heating may also saturate with intensity increase [59]. A more detailed analysis of these processes is out of scope of this paper. The last term in Eq. 2 describes the impact ionization. Due to the strong frequency dependence of this process, see Fig. 4 and Eq. 16, for $\hbar \omega >\varepsilon _{g}$ its contribution is clearly detected for the lowest frequency only, see Fig. 6.

## Summary

To sum up our work, by studying HgTe QWs with nearly linear energy dispersion, we observed that high-power THz radiation results in band-to-band impact ionization. The developed theory, considering the impact ionization for arbitrary values of *ωτ*_*i*_(*ε*), describes the experimental findings well. It shows that in our experiments the light-impact ionization by the electric field of THz radiation is realized. In this case, *ωτ*_*i*_(*ε*) ≫ 1 and the ionization rate depends drastically on the radiation frequency.
